# Prioritizing Asthma Treatment Drugs through Multicriteria Decision Making

**DOI:** 10.1155/2024/6516976

**Published:** 2024-02-05

**Authors:** Sobia Sultana

**Affiliations:** Department of Mathematics and Statistics, Faculty of Science, Imam Mohammad Ibn Saud Islamic University (IMSIU), P.O. Box 90950, Riyadh 11623, Saudi Arabia

## Abstract

Asthma is a medical condition characterized by inflammation, narrowing, and swelling of a person's airways, leading to increased mucus production and difficulties in breathing. Topological indices are instrumental in assessing the physical and chemical attributes of these asthma drugs. As resistance to current treatments continues to emerge and undesirable side effects are linked to certain medications, the search for novel and enhanced drugs becomes a top priority. In this study, the examination of 19 distinct asthma medications was focused. In this study, quantitative structure-activity relationship (QSAR) and quantitative structure-property relationship (QSPR) modeling, in combination with multicriteria decision-making (MCDM) technique VIKOR (VIekriterijumsko KOmpromisno Rangiranje) were employed on asthma drugs, to achieve the most favorable rankings for each asthma drug, taking into account their distinct properties. The topological indices employed for QSPR modeling were Randic index, reciprocal Randic index, Zagreb indices, hyper-Zagreb index, harmonic index, geometric arithmetic index, and forgotten index.

## 1. Introduction

Asthma is an intricate, multifaceted, and chronic noncommunicable disease. Asthma is a heterogeneous disease primarily characterized by inflammation and bronchoconstriction, which causes the airway to narrow [1]. Airflow to the lungs becomes difficult as inflamed airways are more sensitive to environmental cues, producing more mucus. As a result, a person may have an “asthma attack,” characterized by intense coughing, wheezing, pressure in the chest, and breathing issues [2]. Exposure to certain allergens can trigger asthma symptoms [3–5]. Immunoglobulin *E* (IgE) which is allergy-specific responds to irritants and allergens by modulating the production of histamine, tryptase, prostaglandins, and leukotrienes, which constricts the airways. Some nonsteroidal anti-inflammatory medications may also cause the production of mediators, which causes bronchoconstriction [6–8]. Histamine, protease enzymes, tumor necrosis factor (TNF_*α*_), prostaglandins (PG_*s*_), leukotrienes (LT_*s*_), and interleukins (IL_*s*_) are among the chemical mediators implicated in asthma. These are all generated from mast cells, which occur in the lungs and inflammatory cells. Together, these mediators constrict bronchial smooth muscle and produce mucosal oedema, hyperemia, and a discharge of viscid secretions, all of which lead to reversible airway blockage. Inhaled corticosteroids, leukotriene modifiers, and other long-term control drugs for asthma can be divided into two categories: quick-relief medications (bronchodilators), which offer rapid relief during an asthma attack, and long-term control therapies (inhaled corticosteroids, etc.), which assist to reduce inflammation and avoid symptoms. The bronchoconstriction, increased vascular permeability, and eosinophil recruitment caused by the CysLT1 (cysteinyl leukotrienes) receptor are competitively antagonised by the mediator-inhibiting drugs, such as montelukast, pranlukast, and zafirlukast. The drugs ramatroban, setipiprant, and fevipiprant treat the symptoms of asthma by suppressing bronchoconstriction, airway hyper-responsiveness, and inflammatory cell infiltration by antagonistically adhering to the DP2 receptor. Toreforant, an antihistamine, suppresses the effects of histamine, which causes bronchoconstriction and therefore reduces asthma symptoms. Salbutamol, terbutaline, and salmeterol are bronchodilators that stimulate the *β*_2_ receptor. These trigger bronchial muscle cells to release more cAMP, resulting in muscle relaxation [9]. The 2019 coronavirus disease (COVID-19) has emerged as a significant global health concern, particularly for those with preexisting medical issues. Patients dealing with asthma are deemed to be more vulnerable since COVID-19 can seriously impair respiration. Some research suggests that individuals with asthma may be more vulnerable to COVID-19 [10].

Mathematical chemistry is the transformation of mathematical concepts in the research of the chemistry field. The mathematical modeling of chemical mechanisms is part of it. The field of graph theory known as chemical graph theory currently has several applications in the field of chemistry [11]. In theoretical chemistry, chemical compounds are modeled as molecular graphs with vertices and edges. Topological indices play a crucial role in the field of chemical graph theory and computational chemistry, providing valuable insights into the structural characteristics and properties of chemical compounds [12]. These indices, derived from the connectivity of atoms within a molecular structure, offer a quantitative measure of molecular topology without delving into intricate three-dimensional details. By condensing complex molecular structures into numerical values, topological indices facilitate the prediction of various physicochemical, biological, and pharmacological properties of compounds. Researchers leverage these indices for rational drug design, environmental risk assessment, and the elucidation of structure-activity relationships in diverse chemical and biological systems [13–16]. The significance of topological indices lies in their ability to bridge the gap between chemical structure and properties, thereby enhancing our understanding of molecular behavior and aiding in the efficient design and optimization of chemical entities for specific applications.

Quantitative structure-activity and structure-property relationship (QSAR/QSPR) models [17] are the correlation between each numerical descriptor and the attributes related to the referred structure. These models are utilized for the prediction and analysis of the activity, as well as the physical and chemical properties, of chemical structures developed for drugs and materials. This technique partitions a molecule into several numerical concepts called chemical indices [18], each of which independently describes its attributes. First, it has been proven that there is a connection between a chemical compound and a physicochemical or biological property. Next, predictions are made regarding the physicochemical characteristics or biological activities of structurally related compounds [19–27].

Multicriteria decision making (MCDM) [28] is a remarkable field within operational research (OR) [29]. The exploration of diverse objectives using mathematical programming has emerged as a prominent tool for making optimal decisions in specific contexts. There are numerous MCDM methods categorized in different manners. We use the highly compatible VIKOR approach in this research work. Yugoslav researchers Z. S. Jovanovic and M. R. Bozic developed the MCDM method known as VIKOR (VIekriterijumsko KOmpromisno Rangiranje) in 1990 [30]. In a situation when there are several complex criteria to consider when making a decision, the VIKOR technique offers a systematic way to analyze and rank possibilities. It enables decision-makers to pinpoint the most advantageous compromise solution. It considers the simultaneous objectives of increasing the advantages and reducing the disadvantages. Here, I am going to introduce and develop a mathematical connection between OR (operations research) and the biochem graphical sciences. I have created a decision matrix that represents the performance of each alternative concerning each criterion. The VIKOR approach is a practical and simple way to determine the suitable drugs for a patient. Li et al. used an objective approach to handle criteria weighting [31] while applying the VIKOR method to determine optimal rankings for various anticancer drugs, achieving results for properties such as boiling points and enthalpy of vaporization using QSPR. Guoping Zhang et al. ranked specific networks by applying the SAW method and TOPSIS method [32], and they determined criteria weights using the entropy method.

In this study, the optimal ranking will be determined for asthma drugs that have not been previously investigated. There are 19 alternatives (drug structures) as illustrated in Figure 1.1 in Supplementary Materials and 10 attributes (topological indices). First, criteria importance through intercriteria correlation (CRITIC) is employed for the calculation of objective weights assigned to each criterion associated with the selection of asthma drugs. I have also analyzed the correlation between the calculated topological indices and the rankings obtained through the MCDM process. The CRITIC method takes into account the intensity of contrast and conflict within the decision-making problem's structure [33]. These contrasts between criteria are established through correlation analysis [34]. Readers are urged to explore additional techniques for weight computations, such as BCM and best worst criteria [35, 36]. Lastly, VIKOR is used to provide a ranking of potential asthma drugs. In the present investigation, the VIKOR technique optimizes the application of QSPR modeling to rank the targeted asthma drugs most effectively. The findings are derived from the QSPR analysis conducted on 19 effective asthma drugs [37].

The objective of this research work is to compare asthma drugs and identify the most potent ones by evaluating and ranking the most effective drugs for asthma by considering the characteristics (where flash point and boiling point are considered) they exhibit. The major goal of such a study would be to rank asthma treatment medicines in order of importance. Prioritization could be based on efficacy, safety, cost-effectiveness, availability, and patient preferences. The study could help to find the most effective medications or drug combinations for assisting asthma patients in achieving better control over their condition, reducing symptoms, and improving their quality of life. A study of this nature has the potential to enhance the allocation of resources by healthcare systems and policymakers, as it allows for an evaluation of which pharmaceuticals offer the most favorable combination of cost-effectiveness and positive health outcomes.

The research could contribute to the discovery of medications that demonstrate notable efficacy within specific subgroups of asthma patients. Emphasizing drugs that deliver both effectiveness and cost-efficiency has the potential to generate substantial cost reductions for both healthcare systems and patients. Through the identification of optimal treatment choices, the research has the potential to enhance the overall quality of care for asthma patients, with the possibility of lowering hospitalization rates and mitigating adverse consequences linked to inadequately controlled asthma. The study's results may exert an impact on shaping healthcare policies and formulating clinical practice guidelines about asthma treatment, thereby assisting healthcare practitioners in making well-informed decisions.

The rest of this paper unfolds as follows: In the following section, the author will examine fundamental graph theory terms and introduce the degree-based topological indices that are pertinent to our analysis. This emphasizes the importance of investigating the material and the forthcoming research. By calculating these topological indices, researchers may obtain valuable insight into the molecular structure-activity relationships of these drugs. In Section 3, Microsoft Excel is identified as the data analysis tool used to extract results generated by QSPR modeling via regression analysis.  Section 4 discusses the results and the associated discussions. First, the author establishes a framework for integrating QSPR findings into VIKOR analysis. This is achieved by introducing the CRITIC method and providing a detailed explanation of the steps involved in CRITIC. Subsequently, the CRITIC technique is implemented to determine criteria weights. Moreover, both beneficial and nonbeneficial criteria affecting flash point and boiling point are evaluated. Additionally, rankings for flash point and boiling point are presented, and weights are assigned based on correlation coefficients.  Section 5 offers additional discussion and concluding remarks and outlines directions for future research.

## 2. Definitions of Degree-Based Topological Indices of Graph

We consider the molecular graph as an ordered pair, denoted by *ℳ*(*V*(*ℳ*), *E*(*ℳ*)), with a vertex set *V*(*ℳ*) and an edge set *E*(*ℳ*), respectively. The edge set is referred to as linkages between the atoms, while the vertex set is referred to as atoms. Let *p* ∈ *V*(*ℳ*), the number of edges that are incident to a vertex *p*, represented as *ℜ*(*p*), determine its degree (or valency). In this study, connected, simple, finite, and loop-free graphs are utilized specifically.

**Definition 1 .**

Randic´ index was given by Milan Randic´ in [38] and is described as the sum of reciprocals of the square root of the product of vertex degrees of all edges in the graph. Mathematically, it can be represented as follows:

(1) $$\begin{array}{c} R\left(M\right)=\left(\underset{pq\in E\left(M\right)}{\sum }\frac{1}{\sqrt{R\left(p\right)\times R\left(q\right)}}\right). \end{array}$$

**Definition 2 .**

The reciprocal Randic index, represented as RR(*ℳ*), is a modified version of the Randic index that adjusts the calculation by considering the square root of the product of vertex degrees for each edge, rather than using its reciprocal. It was defined by Favaron et al. [39].

The formula for the reciprocal Randic index of a molecular graph *ℳ* is given as follows:

(2) $$\begin{array}{c} \text{RR}\left(M\right)=\left(\underset{pq\in E\left(M\right)}{\sum }\sqrt{R\left(p\right)\times R\left(q\right)}\right). \end{array}$$

**Definition 3 .**

Gutman and Trinajstic´ introduced and defined the first Zagreb index (denoted as *M*_1_(*ℳ*)) as the sum of the vertex degrees for each edge in the graph and the second Zagreb index (denoted as *M*_2_(*ℳ*)) in [40–42] as the sum of the products of the vertex degrees for each edge in the graph. Mathematically, it can be represented as follows:

(3) $$\begin{array}{c} M_{1}\left(M\right)=\underset{pq\in E\left(M\right)}{\sum }\left(R\left(p\right)+R\left(q\right)\right),\\ M_{2}\left(M\right)=\underset{pq\in E\left(M\right)}{\sum }\left(R\left(p\right)\times R\left(q\right)\right). \end{array}$$

**Definition 4 .**

In [43], Shirdel et al. introduced the hyper-Zagreb index and defined it as the sum of the square of the vertex degrees for each edge in the molecular graph. Mathematically, it can be represented as follows:

(4) $$\begin{array}{c} \text{HM}\left(M\right)=\underset{pq\in E\left(M\right)}{\sum }{\left[R\left(p\right)+R\left(q\right)\right]}^{2}. \end{array}$$

**Definition 5 .**

The harmonic index was proposed by Fajtlowicz [44]. It is calculated based on the harmonic mean of the degrees of adjacent vertices in the graph.

The formula for the harmonic index of a graph *ℳ* is given as follows:

(5) $$\begin{array}{c} H\left(M\right)=\underset{pq\in E\left(M\right)}{\sum }\frac{2}{R\left(p\right)+R\left(q\right)}. \end{array}$$

It emphasizes the contribution of less connected vertices to the overall connectivity of the graph. A higher harmonic index value indicates a more complex and well-connected graph structure.

**Definition 6 .**

Vukicevic and Furtula [45] proposed the geometric arithmetic index as the geometric mean and arithmetic mean of the degrees of adjacent vertices in the graph. The formula for the geometric arithmetic index of a molecular graph *ℳ* is given as follows:

(6) $$\begin{array}{c} \text{GA}\left(M\right)=\underset{pq\in E\left(M\right)}{\sum }\frac{2\sqrt{R\left(p\right)\times R\left(q\right)}}{R\left(p\right)+R\left(q\right)}. \end{array}$$

It provides insights into the balance between connectivity and degrees adjacent vertices in a molecular graph.

**Definition 7 .**

Furtula and Gutman [46] introduced the forgotten topological index as the sum of squares of the degrees of vertices of a graph. Mathematically, it can be represented as follows:

(7) $$\begin{array}{c} F\left(M\right)=\underset{pq\in E\left(M\right)}{\sum }\left[{\left(R\left(p\right)\right)}^{2}+{\left(R\left(q\right)\right)}^{2}\right]. \end{array}$$

**Definition 8 .**

Zhou and Trinajstić [47] introduced the sum-connectivity index, denoted as SCI(*ℳ*), which is a measure of the sum of the reciprocal of square roots of the sums of degrees of adjacent vertices in the graph. The formula for the sum-connectivity index of a molecular graph *ℳ* is given as follows:

(8) $$\begin{array}{c} \text{SCI}\left(M\right)=\underset{pq\in E\left(G\right)}{\sum }\frac{1}{\sqrt{R\left(p\right)+R\left(q\right)}}. \end{array}$$

It provides insights into the connectivity patterns within a molecular graph.

**Definition 9 .**

In [48], Estrada et al. introduced and investigated the atom-bond connectivity index (denoted as ABC(*ℳ*)). It is defined as follows:

(9) $$\begin{array}{c} \text{ABC}\left(M\right)=\underset{pq\in E\left(M\right)}{\sum }\frac{\sqrt{R\left(p\right)+R\left(q\right)-2}}{R\left(p\right)\times R\left(q\right)}. \end{array}$$

Atom-bond connectivity index focuses on the contributions of individual bonds to the overall connectivity of a molecular graph.

## 3. Materials and Methods

The data analysis tool of Microsoft Excel is used to obtain results generated from QSPR modeling via regression analysis. All the data in charts and tables evaluated for the VIKOR technique are computed in Microsoft Excel.

## 4. Result and Discussions

### 4.1. Creating a Framework for Incorporating QSPR Findings into VIKOR Analysis

We are evaluating alternative medications for asthma disease based on standardized criteria established during our case study, which aims to generate quantitative structure-property relationship (QSPR) observations. Our goal is to obtain the most optimal outcome as we make our final decision. VIKOR utilizes a ranking system to assess medications for asthma treatment and manages the compromised treatment that closely aligns with the best option.

Step 1: We aim to identify the optimal best *s*_*i*_^+^ and worst *s*_*i*_^−^ values for all criterion functions, denoted as *i*=1,…, n.
For benefit-type functions (i.e., where improvement is desired), the ideal best value *s*_*i*_^+^ is determined as the max{*s*_*ij*_ : *j*=1, 2,…, *J*} while the ideal worst value *s*_*i*_^−^ is determined as the min{*s*_*ij*_ : *j*=1, 2,…, *J*}.
Conversely, for cost-type functions (where higher values are preferred), *s*_*i*_^+^=min{*s*_*ij*_ : *j*=1, 2,…, *J*} while *s*_*i*_^−^=max{*s*_*ij*_ : *j*=1, 2,…, *J*}.
Step 2: Evaluation of *D*_*j*_ (weighted normalized Manhattan distance) and *B*_*j*_ (weighted normalized Chebyshev distance) values.
  (10) $$\begin{array}{c} D_{j}=\overset{m}{\underset{j=1}{\sum }}w_{i}\times \frac{\left({s_{i}}^{+}-s_{ij}\right)}{\left({s_{i}}^{+}-{s_{i}}^{-}\right)},\\ B_{j}=\max\left[w_{i}\times \frac{\left({s_{i}}^{+}-s_{ij}\right)}{\left({s_{i}}^{+}-{s_{i}}^{-}\right)}\right], \end{array}$$
here *w*_*i*_ represents the criteria weights of criteria, *D*_*j*_ represents a decision value associated with a specific decision alternative, indexed by *j*, and *B*_*j*_ represents the maximum value among a set of expressions, each evaluating the performance or desirability of alternative *j* concerning different criteria in a multicriteria decision making (MCDM).
Step 3: Calculation of values *L*_*j*_ for *j*=1, 2,…, *J* is achieved using the following equality:
  (11) $$\begin{array}{c} L_{j}=\nu \times \frac{\left(D_{j}-D^{+}\right)}{\left(D^{-}+D^{+}\right)}+\left(1-\nu \right)\times \frac{\left(B_{j}-B^{+}\right)}{\left(B^{-}+B^{+}\right)}. \end{array}$$
Here, we define *D*^+^=min{*D*_*j*_, *j*=1, 2,…, *J*}, *D*^−^=max{*D*_*j*_, *j*=1, 2,…, *J*}, *B*^+^=min{*B*_*j*_, *j*=1, 2,…, *J*}, and *B*^−^=max{*B*_*j*_, *j*=1, 2,…, *J*}. Additionally, we introduce “ *ν*” as the weighting factor for the strategy of maximizing group utility. Here, (1 − *ν*) represents the weight assigned to individual regret. This strategy may be influenced by a compromise value of *ν*, which could be set at 0.5.
Step 4: We rank the alternatives in ascending order based on the values of *D*, *B*, and *L*, starting with the lowest values.
The alternative with the lowest VIKOR value is identified as the optimal choice. This suggestion aligns closely with the ideal point, as it is ranked best according to the *L* (minimum) measure.

### 4.2. Implementation of the CRITIC (Criteria Importance through Intercriteria Correlation) Technique

Careful consideration is essential when choosing drugs for disease treatment, as it constitutes a significant and crucial decision. Multicriteria decision making (MCDM) is used to address this choice, considering both quantitative and qualitative factors. This section employs CRITIC (criteria importance through intercriteria correlation) methods to address the issue of selecting asthma treatment drugs. The CRITIC method is utilized to determine the weights of the criteria for selecting the most suitable asthma drugs, while the VIKOR approach is employed to generate a comprehensive ranking of the available asthma drug alternatives.

Step 1: The decision matrix A is formed. It shows the performance of different alternatives concerning various criteria.
  (12) $$\begin{array}{c} A={\left[a_{ij}\right]}_{m\times n}=\left[\begin{array}{ccc} a_{11} & a_{12} & \begin{array}{cc} \cdots & a_{1n} \end{array}\\ a_{21} & a_{22} & \begin{array}{cc} \cdots & a_{2n} \end{array}\\ \begin{array}{c} \vdots \\ a_{m1} \end{array} & \begin{array}{c} \vdots \\ a_{m2} \end{array} & \begin{array}{c} \begin{array}{cc} \ddots & \vdots \end{array}\\ \begin{array}{cc} \cdots & a_{mn} \end{array} \end{array} \end{array}\right]\;\left(i=1,2\ldots ,m\;\text{and}\;j\right., \end{array}$$
where *a*_*ij*_ presents the performance value of *i*th alternative on *j*th criterion.
Step 2: The decision matrix is normalized using the following equation:
  (13) $$\begin{array}{c} {a_{ij}}^{*}=\left(\frac{a_{ij}-\;\min\;\left(a_{ij}\right)}{\max\;\left(a_{ij}\right)-\;\min\;\left(a_{ij}\right)}\right)\;i=1,2,\ldots ,m\;\text{and}\;j=1,2,\ldots ,n, \end{array}$$
where *a*_*ij*_^*∗*^ is the normalized performance value of *i*th alternative on *j*th criterion. Here, it should be noted that normalization does not take into account the type of criteria.
Step 3: While determining the criteria weights, both the standard deviation of the criterion and its correlation between other criteria are included. In this regard, the weight of the *j*th criterion (*ω*_*j*_) is obtained as follows:
  (14) $$\begin{array}{c} \left.w_{j}=\frac{C_{j}\;}{\overset{n}{\underset{j=1}{\sum }}C_{j}}\right), \end{array}$$
where *C*_*j*_ is the quantity of information contained in *j*th criterion determined as *C*_*j*_=*σ*_*j*_∑_*j*=1_^*n*^(1 − *r*_*jj*′_), where *C*_*j*_ represents the contribution or importance of the *j*th criterion and *σ*_*j*_ is the standard deviation of the *j* th criterion and *jj*′ is the correlation coefficient between the two criteria. It can be concluded that this method gives the higher weight to the criterion which has high standard deviation and low correlation with other criteria. Specifically, a higher value of *B*_*j*_ indicates that a greater amount of information is obtained from the given criterion, so the relative significance of the criterion for the decision-making problem is higher.

Using the technique mentioned above, we have determined the weights of topological indices, as presented in Table 1, whereas standard error as beneficial and nonbeneficial criteria for both cases shown in Table 2.

I have completed the step-by-step calculations for both Steps 1 and 2, as shown in Tables 1.1 and 1.2, respectively, for flash point. Similar calculations for boiling point are performed in Tables 2.1 and 2.2. Furthermore, the final calculations for Steps 3 and 4, focusing on the investigation of flash point, are included in Table 3, whereas the final calculations for Steps 3 and 4, concentrating on the investigation of boiling point, are included in Table 4.

**Figure 1:**
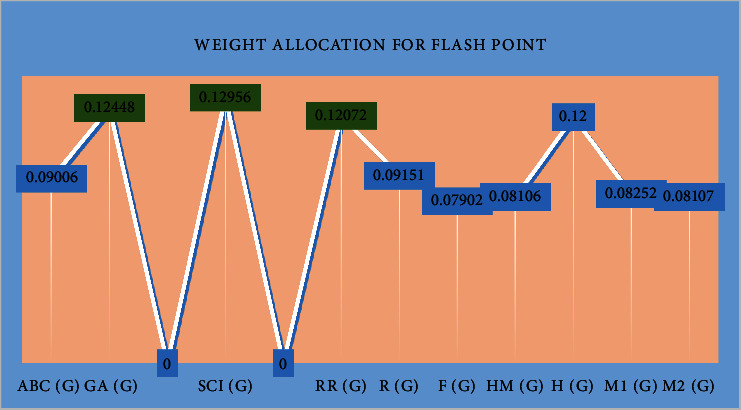
Weight allocation for flash point derived from correlation coefficient.

**Figure 2:**
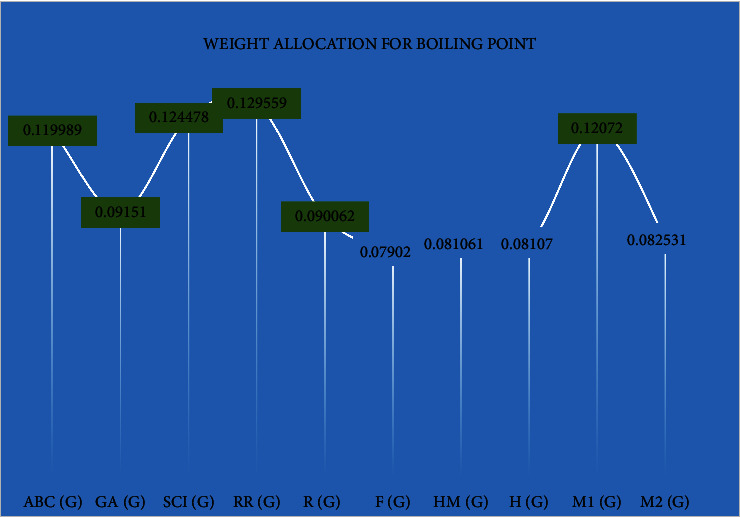
Weight allocation for boiling point derived from correlation coefficient.

It can be seen that fevipiprant is identified as the most suitable drug, considering its proximity to the ideal solution in terms of flash point. On the other hand, montelukast is ranked as the top drug based on boiling point. Weights have been allocated to two criteria, namely, flash point (illustrated in Figure 1) and boiling point (depicted in Figure 2).

Beneficial weights are represented in green color in Figures 1 and 2. Weight allocation for boiling point was derived from correlation coefficient.

**Figure 3:**
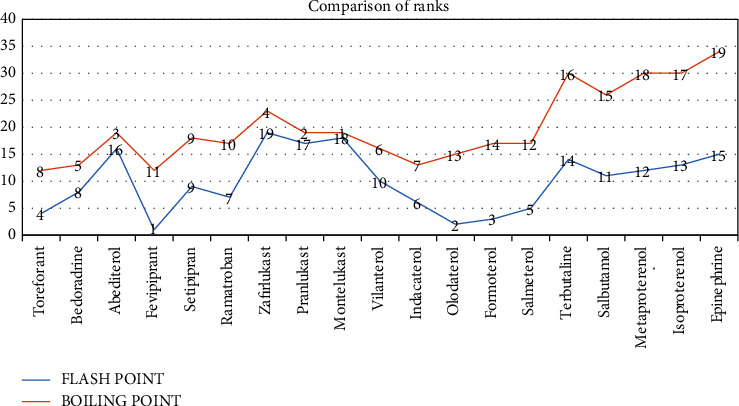
Comparison of ranks according to flash point and boiling point.

The comparison of ranks and drug raking can be seen in Figure 3.

## 5. Conclusions

Priority ranking of drugs required can be viewed as a multicriteria decision-making (MCDM) challenge. This approach has garnered the interest of numerous researchers in recent times. VIKOR is a valuable approach for addressing MCDM problems, and the resulting solution, which is deemed the closest to the ideal solution, is often deemed acceptable to decision-makers. This study suggests using two criteria, namely, boiling point and flash point, to establish the criteria set. The CRTIC method is employed to calculate weights that aid the decision-maker in determining the priority of drug interventions for various utilities.

Nineteen asthma medications were selected to illustrate an application of the proposed highly effective MCDM technique VIKOR method. The VIKOR methodology heavily relies on evaluations and has been applied within the context of QSPR modeling. We conclude that fevipiprant is determined to be the most suitable drug for being closest to the ideal solution taking flash point into account. However, according to the boiling point, montelukast is the ranked one drug. Theoretical findings of this nature could prove valuable for the future ranking of drug structures using chemical invariants, particularly within the domains of biomedicine and mathematical chemistry, facilitating drug discovery endeavors.

Future research in the field of asthma treatment should focus on utilizing real-world data, patient-centered outcomes, and long-term safety evaluations. Comparative studies will help determine the most effective drugs for different patient groups. Precision medicine, machine learning, patient education, and digital health solutions offer the potential for more personalized and effective asthma management.

The proposed approach can be adapted for various medical decision-making challenges, offering the potential as a valuable decision-support tool for asthma treatment. Additionally, this approach can be applied to other diseases in the future.

## Figures and Tables

**Table 1:** Weight determination through the CRITIC method.

Topological indices	Ϭ	Σ (1 − *r*_*jj*′_)	*C*_*j*_	*W*_*j*_
ABC (G)	0.288	11.877	3.424	0.110
GA (G)	0.291	8.177	2.381	0.077
SCI (G)	0.282	8.093	2.280	0.074
RR (G)	0.290	13.881	4.026	0.130
R (G)	0.277	8.0755	2.236	0.072
F (G)	0.301	8.158	2.455	0.079
HM (G)	0.286	8.100	2.317	0.075
H (G)	0.284	13.757	3.911	0.126
M_1_ (G)	0.282	6.107	1.724	0.056
M_2_ (G)	0.284	13.851	3.934	0.127
ABC (G)	0.286	8.107	2.321	0.075

**Table 2:** Beneficial and nonbeneficial criteria impacting flash point and boiling point (BP).

Topological index	Flash point	Boiling point
ABC (G)	0.946	0.970
GA (G)	0.952	0.968
SCI (G)	0.953	0.971
RR (G)	0.952	0.974
R (G)	0.949	0.967
F (G)	0.909	0.941
HM (G)	0.928	0.954
H (G)	0.949	0.964
M_1_ (G)	0.943	0.970
M_2_ (G)	0.945	0.966

**Table 3:** Results for *D*_*j*_, *B*_*j*_, *L*_*j*_, and ranking concerning flash point.

Drugs	*D*	*B*	*L*	Ranks
Toreforant	0.514	0.071	0.351	4
Bedoradrine	0.521	0.078	0.417	8
Abediterol	0.536	0.088	0.516	16
Fevipiprant	0.515	0.061	0.280	1
Setipiprant	0.537	0.076	0.435	9
Ramatroban	0.528	0.069	0.368	7
Zafirlukast	0.624	0.120	0.929	19
Pranlukast	0.565	0.106	0.703	17
Montelukast	0.624	0.119	0.926	18
Vilanterol	0.508	0.085	0.443	10
Indacaterol	0.521	0.071	0.364	6
Olodaterol	0.510	0.063	0.282	2
Formoterol	0.466	0.075	0.284	3
Salmeterol	0.486	0.079	0.354	5
Terbutaline	0.408	0.119	0.490	14
Salbutamol	0.414	0.114	0.468	11
Metaproterenol	0.391	0.122	0.476	12
Isoproterenol	0.391	0.122	0.476	13
Epinephrine	0.375	0.130	0.500	15
	*D* ^*∗*^	0.375	*B* ^*∗*^	0.061
	*D*−	0.624	*B*−	0.130

**Table 4:** Results for *D*_*j*_, *B*_*j*_, *L*_*j*_, and ranking about boiling point (BP).

Drugs	*D*	*B*	*L*	Ranks
Toreforant	0.469	0.056	0.210	8
Bedoradrine	0.453	0.055	0.181	5
Abediterol	0.428	0.058	0.168	3
Fevipiprant	0.450	0.058	0.237	11
Setipiprant	0.455	0.059	0.211	9
Ramatroban	0.476	0.055	0.214	10
Zafirlukast	0.324	0.083	0.172	4
Pranlukast	0.368	0.071	0.165	2
Montelukast	0.323	0.081	0.162	1
Vilanterol	0.440	0.060	0.199	6
Indacaterol	0.475	0.054	0.206	7
Olodaterol	0.497	0.065	0.301	13
Formoterol	0.533	0.085	0.473	14
Salmeterol	0.460	0.070	0.283	12
Terbutaline	0.660	0.124	0.880	16
Salbutamol	0.648	0.121	0.841	15
Metaproterenol	0.666	0.130	0.920	18
Isoproterenol	0.666	0.129	0.920	17
Epinephrine	0.691	0.137	1	19
Toreforant	0.469	0.056	0.210	8
	*D* ^*∗*^	0.323	*B* ^*∗*^	0.054
	*D*−	0.691	*B*−	0.137

## Data Availability

The data used to support the findings of the study are included as Supplementary Materials.

## Supplementary Materials

Supplementary Materials Additional information is provided to enhance the understanding of this study through supplementary figures and tables. Supplementary figure: Figure 1.1: it illustrates the structure of listed asthma drugs used in COVID-19. Supplementary tables: Table 1.1: in this table, the initial input matrix entails the allocation of weights and the classification of chemical indices according to criteria determining their advantages or disadvantages (flash point case). Table 1.2: the calculation of *D*_*j*_ and *B*_*j*_ for flash point is depicted in this table. Table 2.1: in this table, the initial input matrix is displayed, featuring assigned weights and the classification of chemical indices based on criteria for their benefits or nonbenefits (boiling point case). Table 2.2: The calculation of *D*_*j*_ and *B*_*j*_ for boiling point is illustrated in this table. These additional materials provide valuable insights and details that complement and expand upon the main findings discussed in the main text.
