# Supplementary material for: Prioritizing Asthma Treatment Drugs through Multicriteria Decision Making
Source: Int J Anal Chem. 2024 Feb 5;2024:6516976. doi: 10.1155/2024/6516976 (PMC10861281; doi:10.1155/2024/6516976)
Supplement: Supplementary Materials — Additional information is provided to enhance the understanding of this study through supplementary figures and tables. Supplementary figure: Figure 1.1: it illustrates the structure of listed asthma drugs used in COVID-19. Supplementary tables: Table 1.1: in this table, the initial input matrix entails the allocation of weights and the classification of chemical indices according to criteria determining their advantages or disadvantages (flash point case). Table 1.2: the calculation of Dj and Bj for flash point is depicted in this table. Table 2.1: in this table, the initial input matrix is displayed, featuring assigned weights and the classification of chemical indices based on criteria for their benefits or nonbenefits (boiling point case). Table 2.2: The calculation of Dj and Bj for boiling point is illustrated in this table. These additional materials provide valuable insights and details that complement and expand upon the main findings discussed in the main text. [file 6516976.f1.zip › Prioritizing Asthma Treatment Drugs Through MCDM( supplementary file).docx]

**Prioritizing Asthma Treatment Drugs Through Multi-Criteria Decision Making**

**Sobia Sultana**

Department of Mathematics and Statistics, Faculty of Science, Imam Mohammad Ibn Saud Islamic University(IMSIU), P.O. Box 90950, Riyadh 11623, Saudi Arabia;

^*^ Correspondance: Email [ssmahmood@imamu.edu.sa](mailto:ssmahmood@imamu.edu.sa)

**Table 1.1:** Initial input matrix with weights assignments and classifying chemical indices as beneficial and non-beneficial criteria (Flash Point Case).

|  | NB | B | B | B | NB | NB | NB | NB | NB | NB |
| --- | --- | --- | --- | --- | --- | --- | --- | --- | --- | --- |
| Weights | 0.0901 | 0.125 | 0.130 | 0.121 | 0.092 | 0.079 | 0.081 | 0.120 | 0.083 | 0.0811 |
|  | ABC(G) | GA(G) | SCI(G) | RR(G) | R(G) | F(G) | HM(G) | H(G) | M1(G) | M2(G) |
| Toreforant | 22.902 | 31.100 | 14.683 | 75.019 | 13.991 | 394 | 754 | 13.530 | 154 | 180 |
| Bedoradrine | 23.720 | 31.930 | 15.313 | 75.716 | 14.867 | 396 | 754 | 14.300 | 156 | 179 |
| Abediterol | 25.171 | 33.949 | 16.338 | 79.534 | 15.882 | 418 | 792 | 15.352 | 164 | 187 |
| Fevipiprant | 22.836 | 35.175 | 13.861 | 74.619 | 13.392 | 452 | 826 | 12.471 | 158 | 187 |
| Setipipran | 24.115 | 33.181 | 15.435 | 82.221 | 14.525 | 440 | 850 | 14.100 | 168 | 205 |
| Ramatroban | 23.120 | 30.754 | 14.588 | 75.615 | 14.017 | 421 | 791 | 13.386 | 157 | 185 |
| Zafirlukast | 32.271 | 43.495 | 20.546 | 106.524 | 19.640 | 582 | 1102 | 18.886 | 220 | 260 |
| Pranlukast | 28.300 | 39.368 | 18.591 | 92.555 | 17.670 | 464 | 902 | 17.366 | 188 | 219 |
| Montelukast | 32.442 | 43.355 | 20.522 | 105.964 | 19.600 | 588 | 1100 | 18.819 | 220 | 256 |
| Vilanterol | 23.610 | 32.225 | 15.735 | 72.388 | 15.524 | 354 | 682 | 15.100 | 148 | 164 |
| Indacaterol | 22.713 | 31.120 | 14.654 | 76.115 | 13.995 | 406 | 780 | 13.533 | 156 | 187 |
| Olodaterol | 21.738 | 28.723 | 13.721 | 70.010 | 13.270 | 390 | 726 | 12.633 | 146 | 168 |
| Formoterol | 18.632 | 25.201 | 12.232 | 58.317 | 12.028 | 298 | 568 | 11.600 | 120 | 135 |
| Salmeterol | 22.058 | 30.493 | 14.919 | 66.924 | 14.703 | 314 | 610 | 14.433 | 136 | 148 |
| Terbutaline | 12.078 | 14.799 | 7.289 | 36.171 | 7.277 | 218 | 386 | 6.666 | 78 | 84 |
| Salbutamol | 12.635 | 15.896 | 7.827 | 38.404 | 7.832 | 226 | 406 | 7.266 | 82 | 90 |
| Metaproterenol | 11.113 | 14.168 | 6.986 | 33.256 | 6.986 | 180 | 332 | 6.533 | 70 | 76 |
| Isoproterenol | 11.073 | 14.209 | 6.999 | 33.357 | 7.003 | 180 | 334 | 6.566 | 70 | 77 |
| Epinephrine | 9.440 | 12.440 | 6.130 | 28.858 | 6.147 | 152 | 286 | 5.833 | 60 | 67 |
| Ideal Best | 9.440 | 43.495 | 20.546 | 106.524 | 6.968 | 152 | 286 | 6.533 | 60 | 67 |
| Ideal Worst | 32.442 | 14.168 | 6.130 | 33.256 | 19.640 | 588 | 1102 | 18.886 | 220 | 260 |

**Table 1.2:** Computation of D_j_ and B_𝑗_ for Flash Point.

| Toreforant | 0.053 | 0.050 | 0.053 | 0.049 | 0.053 | 0.044 | 0.047 | 0.071 | 0.049 | 0.048 |
| --- | --- | --- | --- | --- | --- | --- | --- | --- | --- | --- |
| Bedoradrine | 0.056 | 0.046 | 0.047 | 0.048 | 0.059 | 0.044 | 0.047 | 0.078 | 0.050 | 0.047 |
| Abediterol | 0.062 | 0.038 | 0.038 | 0.042 | 0.066 | 0.048 | 0.050 | 0.088 | 0.054 | 0.050 |
| Fevipiprant | 0.053 | 0.033 | 0.060 | 0.050 | 0.049 | 0.054 | 0.054 | 0.061 | 0.051 | 0.050 |
| Setipipran | 0.058 | 0.041 | 0.046 | 0.038 | 0.057 | 0.052 | 0.056 | 0.076 | 0.056 | 0.058 |
| Ramatroban | 0.054 | 0.051 | 0.054 | 0.048 | 0.053 | 0.049 | 0.050 | 0.069 | 0.050 | 0.050 |
| Zafirlukast | 0.089 | 0.000 | 0.000 | 0.000 | 0.092 | 0.078 | 0.081 | 0.120 | 0.083 | 0.081 |
| Pranlukast | 0.074 | 0.017 | 0.018 | 0.022 | 0.078 | 0.057 | 0.061 | 0.106 | 0.066 | 0.064 |
| Montelukast | 0.090 | 0.001 | 0.000 | 0.001 | 0.091 | 0.079 | 0.081 | 0.119 | 0.083 | 0.079 |
| Vilanterol | 0.056 | 0.045 | 0.043 | 0.053 | 0.064 | 0.037 | 0.039 | 0.085 | 0.045 | 0.041 |
| Indacaterol | 0.052 | 0.050 | 0.052 | 0.047 | 0.053 | 0.046 | 0.049 | 0.071 | 0.050 | 0.050 |
| Olodaterol | 0.048 | 0.059 | 0.061 | 0.057 | 0.048 | 0.043 | 0.044 | 0.063 | 0.044 | 0.042 |
| Formoterol | 0.036 | 0.073 | 0.075 | 0.075 | 0.040 | 0.027 | 0.028 | 0.053 | 0.031 | 0.029 |
| Salmeterol | 0.049 | 0.052 | 0.051 | 0.062 | 0.058 | 0.029 | 0.032 | 0.079 | 0.039 | 0.034 |
| Terbutaline | 0.010 | 0.115 | 0.119 | 0.109 | 0.008 | 0.012 | 0.010 | 0.008 | 0.009 | 0.007 |
| Salbutamol | 0.013 | 0.111 | 0.114 | 0.106 | 0.011 | 0.013 | 0.012 | 0.013 | 0.011 | 0.010 |
| Metaproterenol | 0.007 | 0.118 | 0.122 | 0.114 | 0.006 | 0.005 | 0.005 | 0.006 | 0.005 | 0.004 |
| Isoproterenol | 0.006 | 0.117 | 0.122 | 0.114 | 0.006 | 0.005 | 0.005 | 0.007 | 0.005 | 0.004 |
| Epinephrine | 0.000 | 0.125 | 0.130 | 0.121 | 0.000 | 0.000 | 0.000 | 0.000 | 0.000 | 0.000 |

**Table 2.1:** Initial input matrix with weights assignments and classifying chemical indices as beneficial and non-beneficial criteria (Boiling Point Case).

|  | B | B | B | B | B | NB | NB | NB | B | NB |
| --- | --- | --- | --- | --- | --- | --- | --- | --- | --- | --- |
| Weights | **0.120** | **0.092** | **0.125** | **0.130** | **0.090** | **0.079** | **0.081** | **0.081** | **0.121** | **0. 083** |
|  | ABC(G) | GA(G) | SCI(G) | RR(G) | R(G) | F(G) | HM(G) | H(G) | M1(G) | M2(G) |
| Toreforant | 22.902 | 31.100 | 14.683 | 75.019 | 13.991 | 394 | 754 | 13.53 | 154 | 180 |
| Bedoradrine | 23.720 | 31.930 | 15.313 | 75.716 | 14.867 | 396 | 754 | 14.3 | 156 | 179 |
| Abediterol | 25.171 | 33.949 | 16.338 | 79.534 | 15.882 | 418 | 792 | 15.352 | 164 | 187 |
| Fevipiprant | 22.836 | 35.175 | 13.861 | 74.619 | 13.392 | 452 | 826 | 12.471 | 158 | 187 |
| Setipipran | 24.115 | 33.181 | 15.435 | 82.221 | 14.525 | 440 | 850 | 14.1 | 168 | 205 |
| Ramatroban | 23.120 | 30.754 | 14.588 | 75.615 | 14.017 | 421 | 791 | 13.386 | 157 | 185 |
| Zafirlukast | 32.271 | 43.495 | 20.546 | 106.524 | 19.640 | 582 | 1102 | 18.886 | 220 | 260 |
| Pranlukast | 28.300 | 39.368 | 18.591 | 92.555 | 17.670 | 464 | 902 | 17.366 | 188 | 219 |
| Montelukast | 32.442 | 43.355 | 20.522 | 105.964 | 19.600 | 588 | 1100 | 18.819 | 220 | 256 |
| Vilanterol | 23.610 | 32.225 | 15.735 | 72.388 | 15.524 | 354 | 682 | 15.100 | 148 | 164 |
| Indacaterol | 22.713 | 31.120 | 14.654 | 76.115 | 13.995 | 406 | 780 | 13.533 | 156 | 187 |
| Olodaterol | 21.738 | 28.723 | 13.721 | 70.010 | 13.270 | 390 | 726 | 12.633 | 146 | 168 |
| Formoterol | 18.632 | 25.201 | 12.232 | 58.317 | 12.028 | 298 | 568 | 11.600 | 120 | 135 |
| Salmeterol | 22.058 | 30.493 | 14.919 | 66.924 | 14.703 | 314 | 610 | 14.433 | 136 | 148 |
| Terbutaline | 12.078 | 14.799 | 7.289 | 36.171 | 7.277 | 218 | 386 | 6.666 | 78 | 84 |
| Salbutamol | 12.635 | 15.896 | 7.827 | 38.404 | 7.832 | 226 | 406 | 7.266 | 82 | 90 |
| Metaproterenol | 11.113 | 14.168 | 6.986 | 33.256 | 6.986 | 180 | 332 | 6.533 | 70 | 76 |
| Isoproterenol | 11.073 | 14.209 | 6.999 | 33.357 | 7.003 | 180 | 334 | 6.566 | 70 | 77 |
| Epinephrine | 9.440 | 12.440 | 6.130 | 28.858 | 6.147 | 152 | 286 | 5.833 | 60 | 67 |
| Ideal Best | 32.442 | 43.495 | 20.546 | 106.524 | 19.640 | 152 | 286 | 6.533 | 220 | 67 |
| Ideal Worst | 9.440 | 14.168 | 6.130 | 33.256 | 6.968 | 588 | 1102 | 18.886 | 60 | 260 |

**Table 2.2:** Computation of D_j_ and B_𝑗_ for Boiling point.

| Toreforant | 0.050 | 0.039 | 0.051 | 0.056 | 0.040 | 0.044 | 0.047 | 0.046 | 0.050 | 0.048 |
| --- | --- | --- | --- | --- | --- | --- | --- | --- | --- | --- |
| Bedoradrine | 0.046 | 0.036 | 0.045 | 0.055 | 0.034 | 0.044 | 0.046 | 0.051 | 0.048 | 0.048 |
| Abediterol | 0.038 | 0.030 | 0.036 | 0.048 | 0.027 | 0.048 | 0.050 | 0.058 | 0.042 | 0.051 |
| Fevipiprant | 0.050 | 0.026 | 0.058 | 0.056 | 0.045 | 0.054 | 0.054 | 0.039 | 0.047 | 0.051 |
| Setipipran | 0.043 | 0.032 | 0.044 | 0.043 | 0.036 | 0.052 | 0.056 | 0.050 | 0.039 | 0.059 |
| Ramatroban | 0.049 | 0.040 | 0.052 | 0.055 | 0.040 | 0.049 | 0.0502 | 0.045 | 0.048 | 0.051 |
| Zafirlukast | 0.001 | 0.000 | 0.000 | 0.000 | 0.000 | 0.078 | 0.081 | 0.081 | 0.000 | 0.083 |
| Pranlukast | 0.022 | 0.013 | 0.017 | 0.025 | 0.014 | 0.057 | 0.061 | 0.071 | 0.024 | 0.065 |
| Montelukast | 0.000 | 0.001 | 0.000 | 0.001 | 0.000 | 0.079 | 0.081 | 0.081 | 0.000 | 0.081 |
| Vilanterol | 0.046 | 0.035 | 0.042 | 0.060 | 0.029 | 0.036 | 0.039 | 0.056 | 0.054 | 0.042 |
| Indacaterol | 0.051 | 0.039 | 0.051 | 0.054 | 0.040 | 0.046 | 0.049 | 0.046 | 0.048 | 0.051 |
| Olodaterol | 0.056 | 0.046 | 0.059 | 0.065 | 0.045 | 0.043 | 0.044 | 0.040 | 0.056 | 0.043 |
| Formoterol | 0.072 | 0.057 | 0.072 | 0.085 | 0.054 | 0.027 | 0.028 | 0.033 | 0.076 | 0.029 |
| Salmeterol | 0.054 | 0.041 | 0.049 | 0.070 | 0.035 | 0.029 | 0.032 | 0.052 | 0.063 | 0.035 |
| Terbutaline | 0.106 | 0.090 | 0.115 | 0.124 | 0.088 | 0.012 | 0.010 | 0.001 | 0.107 | 0.007 |
| Salbutamol | 0.103 | 0.086 | 0.110 | 0.121 | 0.084 | 0.013 | 0.012 | 0.005 | 0.104 | 0.010 |
| Metaproterenol | 0.111 | 0.092 | 0.117 | 0.130 | 0.090 | 0.005 | 0.005 | 0.000 | 0.113 | 0.004 |
| Isoproterenol | 0.112 | 0.091 | 0.117 | 0.129 | 0.090 | 0.005 | 0.005 | 0.000217 | 0.113 | 0.004 |
| Epinephrine | 0.120 | 0.097 | 0.125 | 0.137 | 0.096 | 0.000 | 0.000 | -0.005 | 0.121 | 0.000 |

| 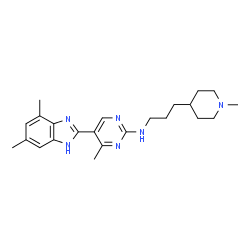 | 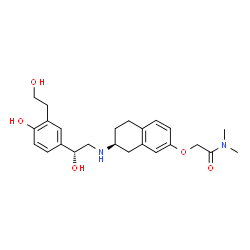 |
| --- | --- |
| (a) Toreforant | (b) Bedoradrin e |
| 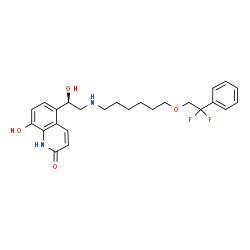 | 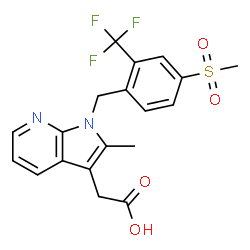 |
| (c) Abeditero l | (d) Fevipiprant |
| 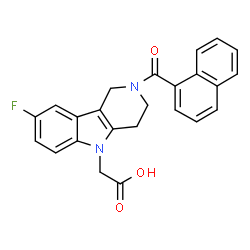 | 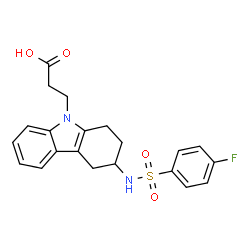 |
| (e) Setipiprant | (f) Ramatroban |
| 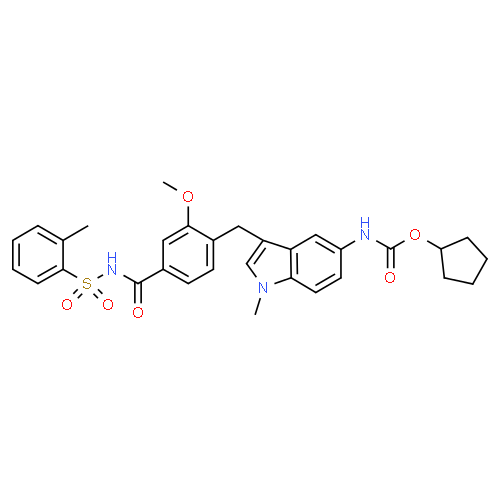 | 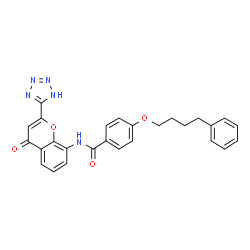 |
| (g) Zafirlukast | (h) Pranlukast |
| 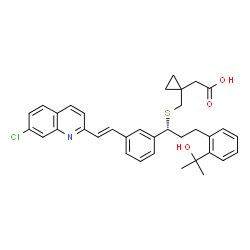 | 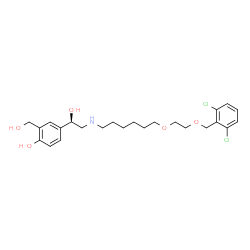 |
| (i) Montelukast | (j) Vilanterol |
| 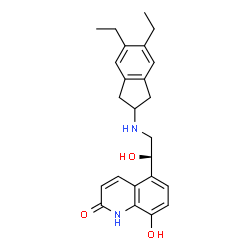 | 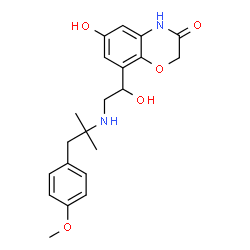 |
| (k) Indacaterol | (l) Olodaterol |
| 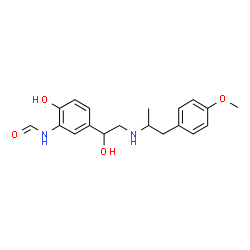 | 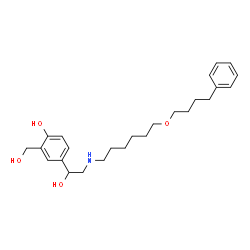 |
| (m) Formoterol | (n) Salmeterol |
| 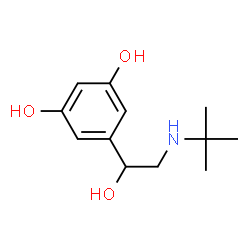 | 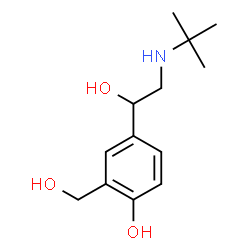 |
| (o) Terbutaline | (p) Salbutamol |
| 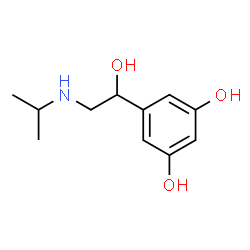 | 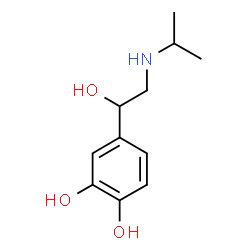 |
| (q) Metaproterenol | (r) Isoproterenol |
| 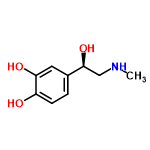 | |
| (s) Epinephrine | |

**Figure 1.1:** Structure of numerous key asthma drugs used in COVID-19.
