# Supplementary material for: Prioritizing Asthma Treatment Drugs through Multicriteria Decision Making
Source: Int J Anal Chem. 2024 Feb 5;2024:6516976. doi: 10.1155/2024/6516976 (PMC10861281; doi:10.1155/2024/6516976)
Supplement: Supplementary Materials — Additional information is provided to enhance the understanding of this study through supplementary figures and tables. Supplementary figure: Figure 1.1: it illustrates the structure of listed asthma drugs used in COVID-19. Supplementary tables: Table 1.1: in this table, the initial input matrix entails the allocation of weights and the classification of chemical indices according to criteria determining their advantages or disadvantages (flash point case). Table 1.2: the calculation of Dj and Bj for flash point is depicted in this table. Table 2.1: in this table, the initial input matrix is displayed, featuring assigned weights and the classification of chemical indices based on criteria for their benefits or nonbenefits (boiling point case). Table 2.2: The calculation of Dj and Bj for boiling point is illustrated in this table. These additional materials provide valuable insights and details that complement and expand upon the main findings discussed in the main text. [file 6516976.f1.zip › Prioritizing Asthma Treatment Drugs Through MCDM( supplementary file).pdf]

Prioritizing Asthma Treatment Drugs Through Multi-Criteria Decision Making

Sobia Sultana

Department of Mathematics and Statistics, Faculty of Science, Imam Mohammad Ibn Saud Islamic University(IMSIU), P.O. Box 90950, Riyadh 11623, Saudi Arabia;

* Correspondance: Email ssmahmood@imamu.edu.sa

Table 1.1: Initial input matrix with weights assignments and classifying chemical indices as beneficial and non-beneficial criteria (Flash Point Case).

	NB	B	B	B	NB	NB	NB	NB	NB	NB
**Weights**	0.0901	0.125	0.130	0.121	0.092	0.079	0.081	0.120	0.083	0.0811
	ABC(G)	GA(G)	SCI(G)	RR(G)	R(G)	F(G)	HM(G)	H(G)	M1(G)	M2(G)
**Toreforant**	22.902	31.100	14.683	75.019	13.991	394	754	13.530	154	180
**Bedoradrine**	23.720	31.930	15.313	75.716	14.867	396	754	14.300	156	179
**Abediterol**	25.171	33.949	16.338	79.534	15.882	418	792	15.352	164	187
**Fevipiprant**	22.836	35.175	13.861	74.619	13.392	452	826	12.471	158	187
**Setipipran**	24.115	33.181	15.435	82.221	14.525	440	850	14.100	168	205
**Ramatroban**	23.120	30.754	14.588	75.615	14.017	421	791	13.386	157	185
**Zafirlukast**	32.271	43.495	20.546	106.524	19.640	582	1102	18.886	220	260
**Pranlukast**	28.300	39.368	18.591	92.555	17.670	464	902	17.366	188	219
**Montelukast**	32.442	43.355	20.522	105.964	19.600	588	1100	18.819	220	256
**Vilanterol**	23.610	32.225	15.735	72.388	15.524	354	682	15.100	148	164
**Indacaterol**	22.713	31.120	14.654	76.115	13.995	406	780	13.533	156	187
**Olodaterol**	21.738	28.723	13.721	70.010	13.270	390	726	12.633	146	168
**Formoterol**	18.632	25.201	12.232	58.317	12.028	298	568	11.600	120	135
**Salmeterol**	22.058	30.493	14.919	66.924	14.703	314	610	14.433	136	148
**Terbutaline**	12.078	14.799	7.289	36.171	7.277	218	386	6.666	78	84
**Salbutamol**	12.635	15.896	7.827	38.404	7.832	226	406	7.266	82	90

**Metaproterenol**	11.113	14.168	6.986	33.256	6.986	180	332	6.533	70	76
**Isoproterenol**	11.073	14.209	6.999	33.357	7.003	180	334	6.566	70	77
**Epinephrine**	9.440	12.440	6.130	28.858	6.147	152	286	5.833	60	67
**Ideal Best**	9.440	43.495	20.546	106.524	6.968	152	286	6.533	60	67
**Ideal Worst**	32.442	14.168	6.130	33.256	19.640	588	1102	18.886	220	260

Table 1.2: Computation of D_j and B_j for Flash Point.

Toreforant	0.053	0.050	0.053	0.049	0.053	0.044	0.047	0.071	0.049	0.048
Bedoradrine	0.056	0.046	0.047	0.048	0.059	0.044	0.047	0.078	0.050	0.047
Abediterol	0.062	0.038	0.038	0.042	0.066	0.048	0.050	0.088	0.054	0.050
Fevipiprant	0.053	0.033	0.060	0.050	0.049	0.054	0.054	0.061	0.051	0.050
Setipipran	0.058	0.041	0.046	0.038	0.057	0.052	0.056	0.076	0.056	0.058
Ramatroban	0.054	0.051	0.054	0.048	0.053	0.049	0.050	0.069	0.050	0.050
Zafirlukast	0.089	0.000	0.000	0.000	0.092	0.078	0.081	0.120	0.083	0.081
Pranlukast	0.074	0.017	0.018	0.022	0.078	0.057	0.061	0.106	0.066	0.064
Montelukast	0.090	0.001	0.000	0.001	0.091	0.079	0.081	0.119	0.083	0.079
Vilanterol	0.056	0.045	0.043	0.053	0.064	0.037	0.039	0.085	0.045	0.041
Indacaterol	0.052	0.050	0.052	0.047	0.053	0.046	0.049	0.071	0.050	0.050
Olodaterol	0.048	0.059	0.061	0.057	0.048	0.043	0.044	0.063	0.044	0.042
Formoterol	0.036	0.073	0.075	0.075	0.040	0.027	0.028	0.053	0.031	0.029
Salmeterol	0.049	0.052	0.051	0.062	0.058	0.029	0.032	0.079	0.039	0.034

Terbutaline	0.010	0.115	0.119	0.109	0.008	0.012	0.010	0.008	0.009	0.007
Salbutamol	0.013	0.111	0.114	0.106	0.011	0.013	0.012	0.013	0.011	0.010
Metaproterenol	0.007	0.118	0.122	0.114	0.006	0.005	0.005	0.006	0.005	0.004
Isoproterenol	0.006	0.117	0.122	0.114	0.006	0.005	0.005	0.007	0.005	0.004
Epinephrine	0.000	0.125	0.130	0.121	0.000	0.000	0.000	0.000	0.000	0.000

Table 2.1: Initial input matrix with weights assignments and classifying chemical indices as beneficial and non-beneficial criteria (Boiling Point Case).

	B	B	B	B	B	NB	NB	NB	B	NB
**Weights**	**0.120**	**0.092**	**0.125**	**0.130**	**0.090**	**0.079**	**0.081**	**0.081**	**0.121**	**0. 083**
	ABC(G)	GA(G)	SCI(G)	RR(G)	R(G)	F(G)	HM(G)	H(G)	M1(G)	M2(G)
**Toreforant**	22.902	31.100	14.683	75.019	13.991	394	754	13.53	154	180
**Bedoradrine**	23.720	31.930	15.313	75.716	14.867	396	754	14.3	156	179
**Abediterol**	25.171	33.949	16.338	79.534	15.882	418	792	15.352	164	187
**Fevipiprant**	22.836	35.175	13.861	74.619	13.392	452	826	12.471	158	187
**Setipipran**	24.115	33.181	15.435	82.221	14.525	440	850	14.1	168	205
**Ramatroban**	23.120	30.754	14.588	75.615	14.017	421	791	13.386	157	185
**Zafirlukast**	32.271	43.495	20.546	106.524	19.640	582	1102	18.886	220	260
**Pranlukast**	28.300	39.368	18.591	92.555	17.670	464	902	17.366	188	219
**Montelukast**	32.442	43.355	20.522	105.964	19.600	588	1100	18.819	220	256
**Vilanterol**	23.610	32.225	15.735	72.388	15.524	354	682	15.100	148	164
**Indacaterol**	22.713	31.120	14.654	76.115	13.995	406	780	13.533	156	187
**Olodaterol**	21.738	28.723	13.721	70.010	13.270	390	726	12.633	146	168

**Formoterol**	18.632	25.201	12.232	58.317	12.028	298	568	11.600	120	135
**Salmeterol**	22.058	30.493	14.919	66.924	14.703	314	610	14.433	136	148
**Terbutaline**	12.078	14.799	7.289	36.171	7.277	218	386	6.666	78	84
**Salbutamol**	12.635	15.896	7.827	38.404	7.832	226	406	7.266	82	90
**Metaproterenol**	11.113	14.168	6.986	33.256	6.986	180	332	6.533	70	76
**Isoproterenol**	11.073	14.209	6.999	33.357	7.003	180	334	6.566	70	77
**Epinephrine**	9.440	12.440	6.130	28.858	6.147	152	286	5.833	60	67
**Ideal Best**	32.442	43.495	20.546	106.524	19.640	152	286	6.533	220	67
**Ideal Worst**	9.440	14.168	6.130	33.256	6.968	588	1102	18.886	60	260

Table 2.2: Computation of D_j and B_j for Boiling point.

Toreforant	0.050	0.039	0.051	0.056	0.040	0.044	0.047	0.046	0.050	0.048
Bedoradrine	0.046	0.036	0.045	0.055	0.034	0.044	0.046	0.051	0.048	0.048
Abediterol	0.038	0.030	0.036	0.048	0.027	0.048	0.050	0.058	0.042	0.051
Fevipiprant	0.050	0.026	0.058	0.056	0.045	0.054	0.054	0.039	0.047	0.051
Setipipran	0.043	0.032	0.044	0.043	0.036	0.052	0.056	0.050	0.039	0.059
Ramatroban	0.049	0.040	0.052	0.055	0.040	0.049	0.0502	0.045	0.048	0.051
Zafirlukast	0.001	0.000	0.000	0.000	0.000	0.078	0.081	0.081	0.000	0.083
Pranlukast	0.022	0.013	0.017	0.025	0.014	0.057	0.061	0.071	0.024	0.065
Montelukast	0.000	0.001	0.000	0.001	0.000	0.079	0.081	0.081	0.000	0.081
Vilanterol	0.046	0.035	0.042	0.060	0.029	0.036	0.039	0.056	0.054	0.042

Indacaterol	0.051	0.039	0.051	0.054	0.040	0.046	0.049	0.046	0.048	0.051
Olodaterol	0.056	0.046	0.059	0.065	0.045	0.043	0.044	0.040	0.056	0.043
Formoterol	0.072	0.057	0.072	0.085	0.054	0.027	0.028	0.033	0.076	0.029
Salmeterol	0.054	0.041	0.049	0.070	0.035	0.029	0.032	0.052	0.063	0.035
Terbutaline	0.106	0.090	0.115	0.124	0.088	0.012	0.010	0.001	0.107	0.007
Salbutamol	0.103	0.086	0.110	0.121	0.084	0.013	0.012	0.005	0.104	0.010
Metaproterenol	0.111	0.092	0.117	0.130	0.090	0.005	0.005	0.000	0.113	0.004
Isoproterenol	0.112	0.091	0.117	0.129	0.090	0.005	0.005	0.000217	0.113	0.004
Epinephrine	0.120	0.097	0.125	0.137	0.096	0.000	0.000	-0.005	0.121	0.000

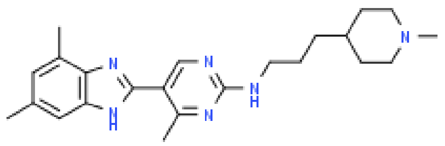

(a) Toreforant

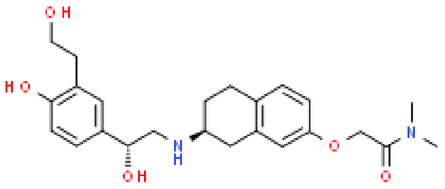

(b) Bedoradrine

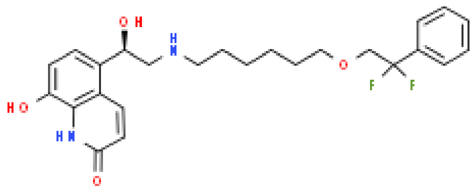

(c) Abediterol

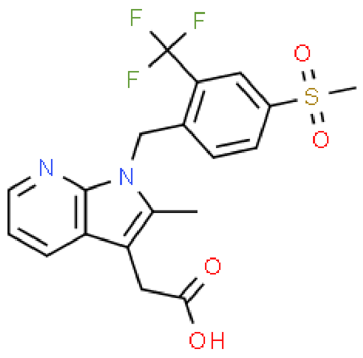

(d) Fevipiprant

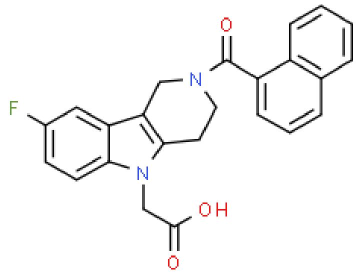

(e) Setipiprant

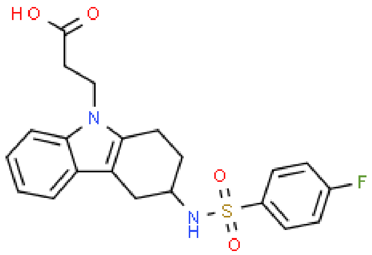

(f) Ramatroban

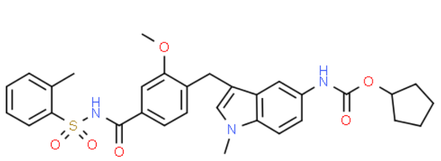

(g) Zafirlukast

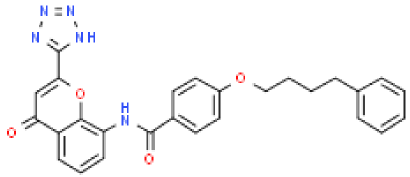

(h) Pranlukast

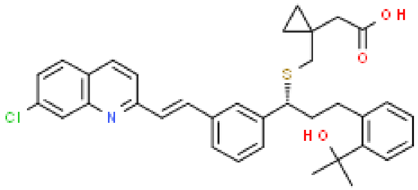

(i) Montelukast

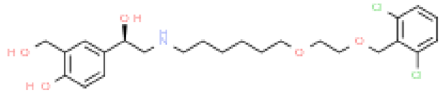

(j) Vilanterol

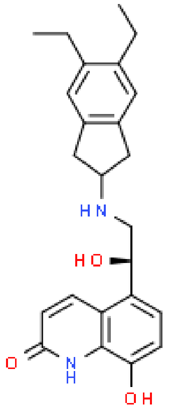

(k) Indacaterol

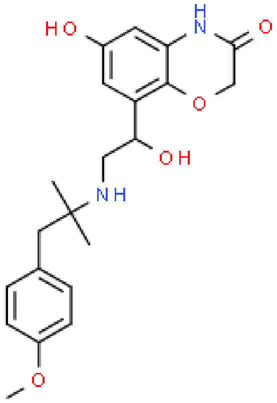

(l) Olodaterol

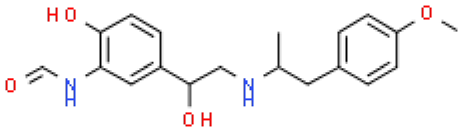

(m) Formoterol

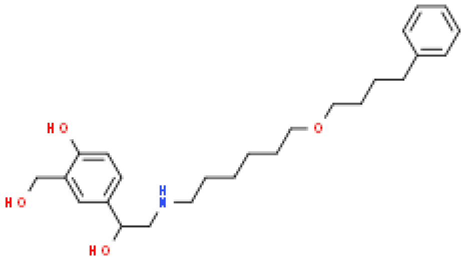

(n) Salmeterol

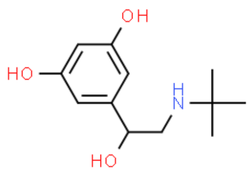

(o) Terbutaline

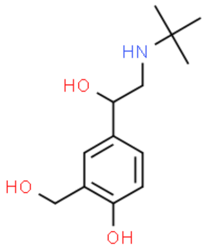

(p) Salbutamol

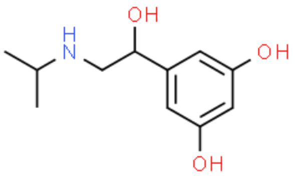

(q) Metaproterenol

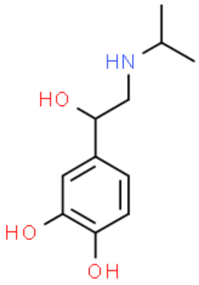

(r) Isoproterenol

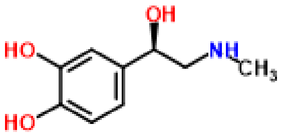

(s) Epinephrine

Figure 1.1: Structure of numerous key asthma drugs used in COVID-19.
